# *Sinorhizobium meliloti* YrbA binds divalent metal cations using two conserved histidines

**DOI:** 10.1042/BSR20202956

**Published:** 2020-10-06

**Authors:** Thomas Roret, Geneviève Alloing, Jean-Michel Girardet, Thomas Perrot, Tiphaine Dhalleine, Jérémy Couturier, Pierre Frendo, Claude Didierjean, Nicolas Rouhier

**Affiliations:** 1 Université de Lorraine, INRAE, IAM, Nancy, France; 2 Sorbonne Universités, CNRS, LBI2M, Roscoff, France; 3 Université Côte d’Azur, INRAE, CNRS, ISA, Sophia Antipolis, France; 4 Institute for Plant Cell Biology and Biotechnology, Heinrich Heine University, Düsseldorf 40225, Germany; 5 Université de Lorraine, CNRS, CRM2, Nancy, France

**Keywords:** bolA, crystallography, glutaredoxin, iron

## Abstract

*Sinorhizobium meliloti* is a nitrogen-fixing bacterium forming symbiotic nodules with the legume *Medicago truncatula. S. meliloti* possesses two BolA-like proteins (BolA and YrbA), the function of which is unknown. In organisms where BolA proteins and monothiol glutaredoxins (Grxs) are present, they contribute to the regulation of iron homeostasis by bridging a [2Fe–2S] cluster into heterodimers. A role in the maturation of iron–sulfur (Fe–S) proteins is also attributed to both proteins. In the present study, we have performed a structure–function analysis of SmYrbA showing that it coordinates diverse divalent metal ions (Fe^2+^, Co^2+^, Ni^2+^, Cu^2+^ and Zn^2+^) using His^32^ and His^67^ residues, that are also used for Fe–S cluster binding in BolA–Grx heterodimers. It also possesses the capacity to form heterodimers with the sole monothiol glutaredoxin (SmGrx2) present in this species. Using cellular approaches analyzing the metal tolerance of *S. meliloti* mutant strains inactivated in the *yrbA* and/or *bolA* genes, we provide evidence for a connection of YrbA with the regulation of iron homeostasis. The mild defects in *M. truncatula* nodulation reported for the *yrbA bolA* mutant as compared with the stronger defects in nodule development previously observed for a *grx2* mutant suggest functions independent of SmGrx2. These results help in clarifying the physiological role of BolA-type proteins in bacteria.

## Introduction

The regulation of metal homeostasis (uptake, cellular and subcellular distribution, incorporation into metalloproteins) is crucial to ensure the proper functioning of numerous biological processes in both eukaryotic and prokaryotic cells. Among the various metals having a physiological role, iron is essential notably because of its incorporation into protein cofactors such as hemes, iron–sulfur (Fe–S) clusters and non-heme mono- or di-iron centers. These moieties will confer to most proteins the capacity to catalyze electron transfer reactions [1]. The regulation of iron homeostasis occurs at different levels (transcriptional or post-transcriptional) and relies on different regulator proteins depending on the model organism considered. A few of these actors may however be shared among organisms and this is the case of BolA and of glutaredoxins (Grxs) belonging to the class II, also referred to as monothiol Grxs [2]. These proteins are found in most organisms and genomic analyses highlighted (i) a strong gene co-occurrence between both protein families, but also (ii) a frequent gene clustering in prokaryote genomes and, (iii) the existence of natural chimeric proteins containing both protein domains in bacteria of the Methylococcales order [3,4]. Accordingly, many high-throughput or targeted studies performed in diverse organisms such as *Saccharomyces cerevisiae, Drosophila melanogaster, Escherichia coli* and *Arabidopsis thaliana* pointed out that the physical interaction between proteins of these families is conserved across kingdoms [5–10]. The study of the biochemical and structural properties of class II Grx and BolA recombinant proteins alone or in complex, showed that both Grx homodimers and Grx–BolA heterodimers bridged [2Fe–2S] clusters and that incubating BolAs with [2Fe–2S]-bridged Grx homodimers promoted their conversions into more stable [2Fe–2S]-bridged BolA–Grx heterodimers [11–19]. Using NMR spectroscopy, it was revealed that Grx and BolA form both apo- and holo-complexes and that the structure of such complexes is primarily driven by electrostatic interactions, involving a putative DNA-binding region in BolAs which adopts a helix-turn-helix structure resembling the one found in K-homology domain proteins [18–22]. For the ligation of the Fe–S clusters in holo-heterodimers, the Grx partner provides cysteine ligands, one from the polypeptide and one from a bound glutathione, whereas the BolA partner provides a histidine ligand, a residue that is totally conserved in the family, and either a second histidine or a cysteine, located in the loop connecting the two first β-strands that was named the [H/C]-loop [13,18,23]. This led to distinguish two subgroups of BolA proteins referred to as BolA_H and BolA_C, respectively [18]. The BolA_H prototypes are found in both prokaryotes and eukaryotes whereas BolA_C prototypes are usually not found in prokaryotes.

In fungi, class II Grxs and BolAs regulate the activity and/or subcellular localization of transcription factors controlling the response to iron starvation/excess i.e., Aft1/2 in *S. cerevisiae*, Php4 and Fep1 in *Schizosaccharomyces pombe*, Cir1 in *Cryptococcus neoformans* or HapX in *Aspergillus fumigatus* [24–27]. In *S. cerevisiae*, the ligation of a [2Fe–2S] cluster in a Fra2/Bol2–Grx3/4 heterodimer regulates the subcellular localization and thus DNA binding activity of Aft1/2 [11,12,15,16,18,25]. It is worth noting that Grx isoforms present in eukaryote organelles have a role in the maturation of Fe–S proteins, owing to their capacity to bridge [2Fe–2S] clusters and transfer them either to proteins from the Fe–S cluster assembly machineries notably ISCA proteins or to client proteins [28–32]. A role of BolA in Fe–S protein maturation is also clearly established in yeast, but it is unclear whether it is connected to the one of Grx. Indeed, the defects observed by deleting the mitochondrial Bol1/3 are different and milder than by deleting Grx5 and indicated an involvement at later maturation stages [20]. These results point to the existence of both common and independent functions of BolA relatively to Grx.

Concerning bacteria, a few information exists about the role of these proteins. Most of them possess a single class II Grx but two BolA_H isoforms referred to as BolA and YrbA/IbaG in *E. coli* [9]. In *E. coli*, BolA was identified as a stress-induced transcriptional regulator capable to modify bacterial cell shape by interacting with promoters of genes encoding proteins involved in the peptidoglycan polymerization machinery and the morphology maintenance systems [33–37]. Data concerning the characterization of YrbA isoforms are scarce but *E. coli* YrbA/IbaG was shown to confer resistance to an acidic stress [38]. Recently, genetic studies in *E. coli* reported an implication of BolA in the maturation of the respiratory complex I [39]. It seems important for complex I stability, likely by participating in the incorporation of the binuclear Fe–S cluster N1b. Noticeably, complex II activity is affected in a double *bolA ibaG* mutant whereas the one of complex I remained as in the single *bolA* mutant. Overall, this suggested that BolA-type proteins in *E. coli* are not strictly interchangeable, but they may have similar functions/Fe–S protein targets as in the case of complex II subunits. This role in Fe–S protein maturation seems consistent with the capacity of *E. coli* BolA and YrbA/IbaG to form [2Fe–2S]-bridging heterodimers with Grx4 [16,23].

In *Sinorhizobium meliloti*, deletion of *grx2*, coding for the sole class II Grx, leads to an impaired activity of the Fe–S containing aconitase and succinate dehydrogenase, to an increased intracellular iron content and to the deregulation of some genes controlled by the major transcriptional regulator of iron homeostasis RirA (rhizobial iron regulator) [40]. This suggests a direct role in the maturation of Fe–S proteins, but possibly other functions such as the regulation of the RirA regulator that senses the cellular iron and O_2_ status through the conversion of a fragile [4Fe–4S] cluster into a [3Fe–4S] cluster [41–43]. One of the consequences of *grx2* deletion was a defect in the development of symbiotic nodules with *Medicago truncatula*, thus impairing nitrogen fixation [40]. As most bacteria, *S. meliloti* possesses two genes encoding BolA proteins that we named SmBolA and SmYrbA by analogy with *E. coli* orthologs. To decipher whether SmYrbA has a role in the regulation of iron homeostasis in *S. meliloti*, we have combined biochemical, biophysical, and structural approaches to investigate the capacity of SmYrbA to interact with SmGrx2 and to bind metal ions. Moreover, we have investigated its physiological role by examining the effects of *yrbA* and *bolA* deficiency on the growth and metal resistance of free-living *S. meliloti* and on the development and function of symbiotic nodules formed with *M. truncatula*.

## Experimental

### Expression and purification of recombinant proteins

The sequences coding for full-length SmGrx2 (SM2011_c00538) and SmYrbA (SM2011_c00487) proteins were cloned by PCR amplification from genomic DNA of the *S. meliloti* Sm2011 strain into the pET-3d and pET-12a or pET-15b expression vectors, respectively (primers listed in Supplementary Table S1). Expression was performed in the *E. coli* BL21 (DE3) strain, containing the pSBET plasmid, that allows expression of the tRNA recognizing AGG and AGA codons. Protein expression was initiated by adding 100 µM IPTG at 37°C for 4 h when cells grown in Luria–Bertani medium supplemented with 50 µg.ml^−1^ ampicillin and kanamycin are in the exponential phase. For untagged proteins, the cell pellets were resuspended in 20 ml of 30 mM Tris-HCl pH 8.0 buffer containing 1 mM EDTA and 200 mM NaCl and stored at −20°C. Cell lysis was performed by sonication (2 × 1 min) and the soluble and insoluble fractions were separated by centrifugation (25 min, 4°C and 48000×***g***). The protein fraction precipitating between 40 and 80% of the saturation contained the recombinant proteins (SmGrx2, SmYrbA) as estimated by 15% SDS/PAGE. Proteins were purified by size exclusion chromatography after loading on Ultrogel™ AcA-44 column equilibrated in a 30 mM Tris-HCl pH 8.0 buffer containing 1 mM EDTA and 200 mM NaCl. The fractions that contained the proteins were pooled, dialyzed by ultrafiltration to remove NaCl, and loaded on a DEAE-cellulose column equilibrated with a 30 mM Tris-HCl pH 8.0, 1 mM EDTA buffer. Proteins were eluted using a 0 to 400 mM NaCl gradient prepared in the same buffer. Recombinant proteins were then dialyzed against a 30 mM Tris-HCl pH 8.0 buffer and concentrated by ultrafiltration under nitrogen pressure (Amicon YM5 membrane) and stored at −20°C. For purification of the N-terminal His-tagged SmYrbA (expressed using pET-15b), the cell pellet was resuspended in 20 ml of a 30 mM Tris-HCl pH 8.0, 300 mM NaCl, 10 mM imidazole buffer and stored at −20°C. Cell lysis was performed by sonication (2 × 1 min) and the soluble and insoluble fractions were separated by centrifugation (25 min, 4°C, 48000×***g***). The purification of His-tagged SmYrbA was performed using an immobilized metal ion affinity chromatography (Ni-NTA Agarose gel; QIAGEN) from the soluble fraction. After cell extract loading, the column was washed with the same buffer and the protein eluted using a 30 mM Tris-HCl pH 8.0, 300 mM NaCl, 250 mM imidazole buffer. After sample concentration, salts were removed by desalting on a Sephadex™ G-25 column. The protein was finally concentrated using Vivaspin® 500 centricon centrifugal filters in 30 mM Tris-HCl pH 8.0 buffer. The protein purity was checked by SDS/PAGE and protein concentrations were determined using a bicinchoninic acid (BCA) assay kit and the provided bovine serum albumin solution for establishing the standard curve (INTERCHIM).

### Analytical size exclusion chromatography

Analytical size exclusion chromatographies were performed using a Superdex™ 75 10/300 column (GE Healthcare) connected to an ÄKTA-Purifier™ (GE Healthcare) at a temperature of 10°C. The column was calibrated with molecular mass standards ranging from 6.5 to 66 kDa (Sigma). The molecular mass of each protein and their elution volume are as follows: aprotinin (bovine lung) - 6500 Da - 14.99 ml; cytochrome *c* (horse heart) - 12400 Da - 12.86 ml; carbonic anhydrase (bovine erythrocytes) - 29000 Da - 11.12 ml, albumin (bovine serum) - 66000 Da - 9.06 ml. A total of 100–200 µg of SmYrbA and SmGrx2 alone or in complex were loaded on the column equilibrated with a buffer containing 30 mM Tris-HCl buffer pH 8.0 and 1 mM DTT at a flow rate of 0.8 ml.min^−1^. The absorbance of the eluted fractions was recorded at 280 nm.

### Crystallization, data collection, structure determination and refinement

Sparse matrix screening was carried out using the microbatch under oil method at 4°C. SmYrbA (38 mg.ml^−1^) crystals were obtained from the Jena Bioscience Crystal Screen 10 condition B1 (100 mM Tris-HCl pH 8.5 buffer, 1 M lithium sulfate and 10 mM nickel chloride). Upon optimization, crystals were also obtained in 100 mM Tris-HCl pH 8.5 buffer containing 0.7 M lithium sulfate and 20 mM copper acetate or 20 mM cobalt chloride using SmYrbA at 19 mg.ml^−1^. Crystals were soaked in mother liquor and further cryoprotected by supplementation with 20% glycerol and frozen in liquid N_2_. X-ray diffraction experiments were performed at 100 K at beamline FIP-BM30A (European Synchrotron Radiation Facility, Grenoble, France). Datasets at 0.80, 0.98 and 1.90 Å for SmYrbA-Cu, SmYrbA-Ni and SmYrbA-Co, respectively, were indexed and processed using XDS [44] and scaled and merged with Scala [45] from the CCP4 program package [46]. The SmYrbA-Ni structure was solved by multiple-wavelength anomalous diffraction (MAD) phasing (Table 1) with AutoSol and AutoBuild from PHENIX [47,48]. SmYrbA-Co and SmYrbA-Cu structures were solved by molecular replacement with Molrep using SmYrbA-Ni coordinates as a template. X-ray crystal structures were refined (Table 1) applying automatic calculations from PHENIX [53] with manual inspection and corrections using Coot [49]. The validation of the crystal structures was performed with MolProbity [50].

**Table 1:** Crystallographic data statistics for SmYrbA–metal complexes

*Data collection*	Peak	Inflection	Remote	SmYrbA-Ni	SmYrbA-Cu	SmYrbA-Co
Beamline	FIP-BM30A					
Space group	*P*2_1_2_1_2_1_					
Average unit cell (Å) *a*,*b*,*c*	30.9; 31.9; 62.8	30.9; 31.9; 62.8	30.9; 31.9; 62.8	30.9; 31.9; 62.8	30.7; 32.2; 62.6	30.8; 31.9; 62.7
Wavelength (Å)	1.4860	1.4866	1.4826	0.9800	0.9800	0.9800
Resolution (Å)	31.9–1.5 (1.6–1.5)	31.9–1.5 (1.6–1.5)	31.9–1.5 (1.6–1.5)	31.9–1.0 (1.03–0.98)	32.2–0.8 (0.84–0.80)	31.9–1.9 (2.0–1.9)
*R*_merge_ (%)	6.6 (22.3)	5.1 (24.8)	4.7 (32.2)	7.4 (35.9)	8.4 (77.8)	14.2 (44.7)
*R*_meas_ (%)	7.2 (26.8)	5.5 (30.0)	5.2 (38.6)	7.7 (43.3)	9.1 (84.2)	15.3 (48.7)
*R*_pim_ (%)	2.8 (14.2)	2.2 (16.1)	2.0 (20.4)	2.3 (23.6)	3.4 (31.9)	5.4 (18.6)
No. unique reflections	9931 (1106)	9961 (1102)	10014 (1125)	35397 (4201)	66196 (9504)	5140 (643)
Mean *I*/σ(*I*) *	16.6 (4.6)	20.9 (4.7)	21.3 (3.9)	22.5 (3.2)	14.8 (2.7)	15.0 (5.7)
CC_1/2_	1.00 (0.96)	1.00 (0.96)	1.00 (0.93)	1.00 (0.82)	1.00 (0.75)	1.00 (0.89)
Completeness (%)	95 (75)	95 (74)	96 (76)	97 (81)	100 (100)	98 (89)
Average redundancy	6 (4)	6 (3)	6 (4)	9 (3)	7 (7)	8 (6)
Anomalous completeness (%)	94 (68)	94 (68)	95 (70)			
Anomalous multiplicity	3 (2)	3 (2)	3 (2)			
*Refinement*						
Resolution (Å)				31.9–1.0	32.2–0.8	31.9–1.9
*R*_free_/*R*_work_ (%)				11.3/10.1	13.2/12.2	15.0/13.3
Total number of atoms				1362	1304	1192
Water				112	150	101
Average B factor (Å^2^)				8.61	9.16	12.85
Ligands				Ni; Li	Cu; Li	Co
*R.m.s deviations*^†^						
Bonds (Å)				0.009	0.032	0.012
Angles (°)				1.372	0.898	1.288
*MolProbity analysi*s						
Clashscore, all atoms				3.2 (86%)	2.6 (88%)	7.3 (91%)
MolProbity score				1.1 (94%)	1.1 (95%)	1.4 (97%)
Ramachandran *analysis*						
Favoured (%)				99	99	99
Allowed (%)				1	1	1
Outliers (%)				0	0	0
PDB entry				5NFK	5NFM	5NFL

Values in parentheses refer to the outer resolution shell. Five percent of reflections were selected for *R*_free_ calculation. *I is the mean intensity, σ(I) is the standard deviation of reflection intensity I. ^†^r.m.s.deviations, root-mean-square deviations of bond length or bond angle.

Excitation scans were performed on SmYrbA-Co, -Cu and -Ni crystals using an energy of 17 keV. The fluorescence counts were recorded with a Roentec X-Flash multi-channel analyzer on beamline FIP-BM30A (European Synchrotron Radiation Facility, Grenoble, France).

### Isothermal titration calorimetry measurements

All isothermal titration calorimetry (ITC) data were collected using a MicroCal iTC200 calorimeter (Malvern Instruments, Malvern, U.K.), with stirring at 1000 rpm, at 20°C and reference power of 5 µcal.s^−1^. First, SmGrx2 (50 µM in 30 mM phosphate buffer pH 8.0) was titrated with SmYrbA (800 µM). Second, SmYrbA (50 µM) used as ligand was titrated with different metal ions (CaCl_2_, CoCl_2_, Cu acetate, FeCl_3_, FeSO_4_, NiCl_2_, and Zn acetate) at 1 mM in 30 mM Tris-HCl buffer pH 8.0. To prevent Fe^2+^ oxidation during binding experiments, both SmYrbA and FeSO_4_ solutions were prepared anaerobically (O_2_ ≤ 1 ppm) within a Jacomex glovebox in a degassed 30 mM Tris-HCl pH 8.0 buffer. The titrant was sequentially injected into the reaction cell (injections of 2 µl for Ni^2+^, Co^2+^, Cu^2+^ and Zn^2+^ and of 1 µl for SmYrbA/SmGrx2, Fe^3+^, Fe^2+^ and Ca^2+^) and the integrated heat data were fit to the one-set-of-sites model in Origin (MicroCal Data Analysis software) according to the manufacturer’s instructions. Experiments were carried out in duplicate and a blank titration (buffer into buffer) was subtracted.

### Real-time analyses of biomolecular interactions

The interactions of SmYrbA with SmGrx2 or with CaCl_2_, CoCl_2_, Cu acetate, NiCl_2_ and Zn acetate were investigated using an electro-switchable DNA chip MPC-48-2-R1-S put in a biosensor analyzer switchSENSE® DRX (Dynamic Biosensors GmbH, Planegg, Germany) according to [51]. For protein–metal ion interaction study, covalent conjugate of SmYrbA and 48mer ssDNA was prepared with the amine coupling kit (Dynamic Biosensors GmbH, Planegg, Germany) and purified by anion-exchange chromatography on to an ÄKTA-Start™ system (GE Healthcare, Chicago, U.S.A.). The purified conjugate was diluted in 10 mM sodium 4-(2-hydroxyethyl)-1-piperazineethanesulfonate (HEPES) pH 7.4 buffer, containing 40 mM NaCl to a final concentration of 200 nM and injected (25 µl) into the chip to form fluorescent SmYrbA-dsDNA nanolevers. To determine affinity constants (*K*_d_ = *k*_off_*/k*_on_) for SmYrbA–metal complexes, serial dilutions of each metal ion prepared at 100 µM in HEPES buffer were injected in the microfluidic at a flow rate of 1 ml.min^−1^ for 20 s (association kinetics, association rate constant (*k*_on_)) and HEPES buffer was then injected at the same flow rate for 20 s (dissociation kinetics, dissociation rate constant (*k*_off_)) at 25°C. The quenching of fluorescence due to the presence of metal ions at the proximity of the Cy5 fluorophore (nanolevers without bound SmYrbA) was subtracted to normalize the signal. For the SmYrbA–SmGrx2 interaction study, the covalent conjugate used was a His-tagged SmYrbA bound to a nickel tris-nitrilotriacetic acid (Ni-NTA_3_)-tagged 48mer DNA. To prevent unspecific binding of His-tagged SmYrbA on to the dsDNA, the protein was injected in the presence of 100 mM NaCl and multiple washing steps using a 10 mM potassium phosphate buffer pH 7.4, containing 2 mM MgCl_2_ to ensure DNA stability were performed. Association kinetics in the switching dynamic mode were determined by injecting SmGrx2 at 25, 50 and 100 µM with a flow rate of 5 µl.min^−1^ for 5 min and dissociation kinetics at 50 µl.min^−1^ for 10 min (at 25°C). All curves were analyzed by nonlinear fitting of single-exponential functions with the switchANALYSIS® software from Dynamic Biosensors GmbH, Planegg, Germany [51].

### Circular dichroism spectroscopy

The circular dichroism (CD) spectra have been recorded using a Chirascan Plus spectropolarimeter (Applied Photophysics, Ltd, U.K.). The content in secondary structures of SmYrbA (81 µM) with or without 3 mM copper acetate was calculated in the far-UV CD region (180–260 nm) in water at 25°C using the software CDNN [52]. Three spectra were averaged, and the results were presented as Δε values (M^−1^.cm^−1^) calculated using Pro-Data Viewer software (Applied Photophysics). For analyzing changes upon SmYrbA–SmGrx2 interaction, quartz spare split-compartment cuvettes with a 0.437 cm path length per compartment were used. The measurements were carried out at 20°C using 10 µM SmYrbA and SmGrx2. The far-UV (200–260 nm) spectra (bandwidth of 1 nm) were recorded with 30 mM phosphate pH 8.0 buffer in the other compartment. Then, each protein prepared in the same buffer was added to each compartment of the cuvette and CD spectra were recorded before and after mixing the cuvette contents. Three spectra were averaged, and the results were presented as Δε values (M^−1^.cm^−1^ per residue).

### Constructions of *S. meliloti bolA* and *yrbA* mutants

*S. meliloti bolA* and *yrbA* single and double mutants were constructed by inserting an antibiotic-resistance cartridge in one or both open reading frames (SM2011_c00698 and SM2011_c00487). First, 1-kb fragments covering the entire gene sequence and the upstream and downstream flanking regions were amplified by PCR with the primer pairs bolA-F GGAGAGGCCGGAAAAATAGT/bolA-R TGAAGAACCGGATCACCAAG and yrbA-F GAGGACGGCCTGGTTACG/yrbA-R CGGGATGAGGCTTAGAACAC for *bolA* and *yrbA* genes, respectively, and cloned in pGEM-T. For the *bolA* single mutant, the 1-kb fragment was subcloned between the NcoI and PstI sites of the pSUP202 suicide-vector [53], then the kanamycin resistance cartridge from pKOK5 was inserted into the EcoRI site of *bolA* sequence. For the *yrbA* single mutant, a PvuII-PstI fragment carrying the kanamycin resistance cartridge was first inserted into the EcoRV site of the *yrbA* sequence, then the whole fragment (ZraI-NdeI blunt) was inserted into the ZraI site of pSUP202. For the *bolA yrbA* double mutant, a spectinomycin resistance cartridge from pHP45-Ω was inserted into the EcoRV site of the *yrbA* sequence and the full fragment was subcloned between the AatII-PstI sites of pSUP202.

The recombinant suicide plasmids were transferred into the *S. meliloti* wildtype Rm2011 recipient strain by biparental mating with *E. coli* S17-1 as a donor strain, as previously described [54]. The double-recombinant clones resulting from the insertion of the antibiotic resistance cartridge inside *bolA* and *yrbA* genes were selected as Nmr and Tcs (*bolA* and *yrbA* single mutants) or Nmr, Spcr and Tcs (*bolA yrbA* double mutant) on M9 minimal medium with sucrose as the carbon source to prevent growth of the *E. coli* donor strain. The recombination events were controlled by PCR.

### Impact of metal ions on bacterial growth

*S. meliloti* Rm2011 and its derivatives were grown at 30°C in LB medium supplemented with 2.5 mM MgSO_4_ and 2.5 mM CaCl_2_ (LBMC) in the presence of appropriate antibiotics, or in M9 mineral salt medium [55] containing 0.4% mannitol as the carbon source. For growth assays in the presence of metals, bacteria were first incubated overnight in LBMC, then washed two times and resuspended in M9 to an OD_600_ of ∼5, and finally diluted 1:100 in M9 with or without metal addition. Iron, cobalt, copper and nickel stock solutions were prepared with FeCl_3_, CoCl_2_, CuCl_2_ and NiCl_2_ in 0.8 M Tris-HCl pH 8.0. The concentrations used in growth assays for each metal (Fe (1 mM), Cu (0.5 mM), Ni (0.5 mM) and Co (0.5 mM)) are the minimal concentrations affecting bacterial growth in minimal medium as determined by preliminary experiments using various concentrations of metals. Bacterial growth was monitored by measuring the optical densities of culture tubes at 600 nm. Data are representative of three independent experiments.

### Plant nodulation and nitrogen fixation assays

*M. truncatula* Jemalong J5 was used as the host plant for assessing nodulation and nitrogen fixation by the *S. meliloti* strains. Plants and bacteria were grown as previously described [40]. For competition experiments, plants were inoculated with a 1:1 mixture of wt and mutant strains (OD_600_ = 0.01). To determine the relative proportion of nodules occupied by mutant or wt bacteria, the wt strain contained a plasmid expressing tetracycline resistance (pXLGD4). Nodules located on the primary roots (88 nodules from 18 plants tested) were manually removed, surface sterilized (1 min exposure to 1% sodium hypochlorite) and rinsed in sterile distilled water. Individual nodules were then crushed in 100 µl sterile distilled water, and 10 µl of each nodule extract were spotted on to LBMC agar plates containing the appropriate antibiotics for strain differentiation. The mean value of the percentage of nodule occupancy was calculated. Data were evaluated for statistical significance using binomial test. Acetylene reduction assay (ARA) was conducted 3 weeks after inoculation, by *in vivo* measurements of acetylene reduction to ethylene, as previously described [56]. The results are presented as acetylene reduction per mg of nodules. Experiments were performed in biological triplicate with 18 nodulated roots per replicate. The significance of differences was assessed with a Student’s *t* test.

## Results and discussion

### Metal-bound SmYrbA structures

**Figure 1:**
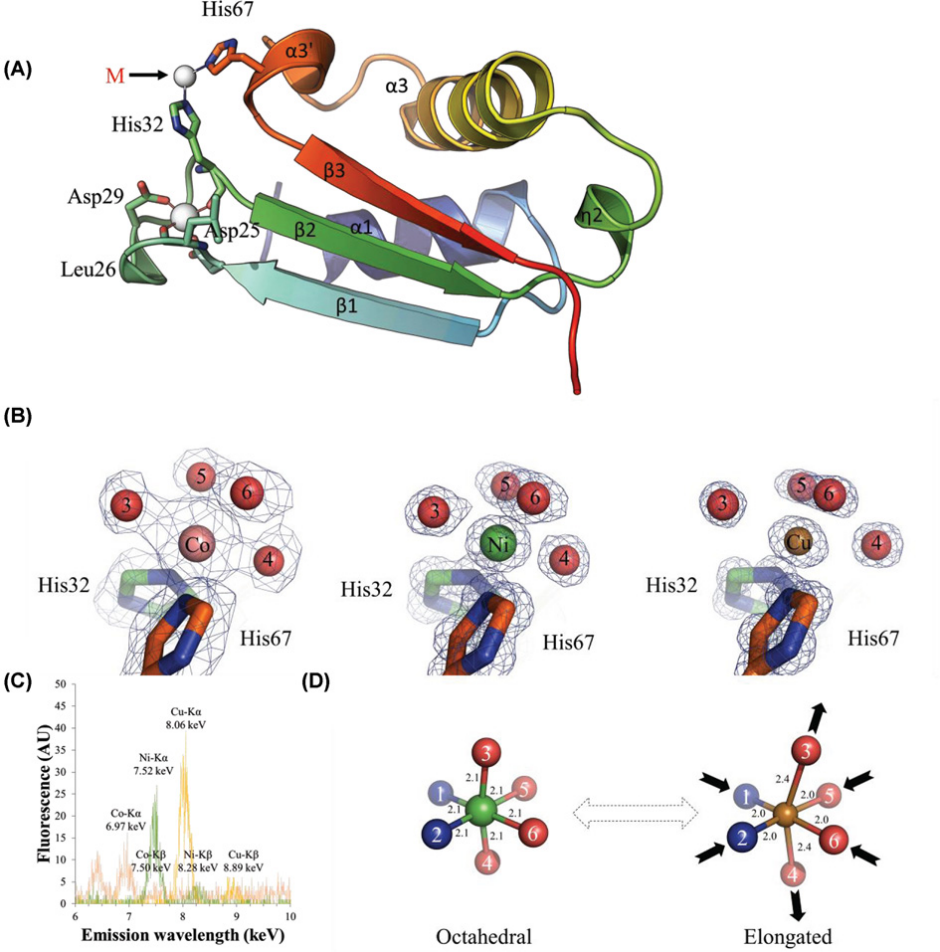
SmYrbA structure and metal binding sites (**A**) SmYrbA crystal structure. It exhibits an α/β-topology composed of three β-strands and four helices as shown by the cartoon representation. There are two metal-binding sites pointed by spheres, one is filled by lithium and the other occupied by Ni, Co or Cu is referred to as M. Residues involved in metal binding are modeled as sticks: Asp^25^, Leu^26^, Asp^29^ for lithium and His^32^ and His^67^ for the other metals. (**B**) Electron density around the cobalt, nickel and copper binding sites (from left to right) in SmYrbA X-ray structures. SmYrbA metal binding site involved His^32^ and the invariant His^67^ residues (shown as sticks). The metal ion and water molecules are shown as spheres. The maps shown are σ_A_-weighted *2mF_o_–DF_c_* maps contoured at 1.2σ (0.62, 0.82 and 1.04 e.Å^−3^, for SmYrbA–Co, SmYrbA–Ni, and SmYrbA–Cu, respectively). (**C**) X-ray fluorescence spectra of SmYrbA crystals. The energies of the emitted fluorescence for SmYrb–Co, SmYrbA–Ni and SmYrbA–Cu are colored pink, green and orange, respectively. Characteristics Kα and Kβ X-ray emission spectra are observed for copper (8.06 and 8.89 keV), nickel (7.52 and 8.28 keV) and cobalt (6.97 and 7.50 keV) in SmYrbA–Cu, SmYrbA–Ni and SmYrbA–Co X-ray structures, respectively. (**D**) Jahn–Teller effect. In the octahedral complex, the nickel atom is equidistant from its ligands. In the case of the copper atom, the octahedral complex is distorted. The distortion takes the form of elongating the axial bonds between copper and the water molecules numbered 3 and 4. The other ligands are a little bit closer to the copper atom compared with the nickel coordination.

To analyze the biochemical and structural properties of SmYrbA, the corresponding recombinant protein was expressed in *E. coli* and purified to homogeneity. Suitable X-ray diffracting crystals were obtained in a crystallization solution containing nickel chloride. Thus, a MAD experiment was attempted at the absorption edge of the nickel atom (Z = 28). We successfully phased the 0.98 Å resolution data with one Ni atom per monomer. This first structure was named SmYrbA-Ni (Figure 1A). Two additional crystal structures have been solved, i.e. SmYrbA–Co complex at 1.90 Å resolution and SmYrbA–Cu complex at an ultra-high resolution of 0.80 Å by replacing nickel chloride by cobalt chloride and copper acetate in the crystallization solution, respectively. All the complexes crystallized in the same space group *P*2_1_2_1_2_1_ with one monomer per asymmetric unit. The calculated low solvent rate of 37% could explain the high diffraction power of the crystals of SmYrbA–Ni and SmYrbA–Cu complexes. The three solved structures are almost identical with a root-mean-square deviation (r.m.s.d.) of atomic positions of 0.096 Å.

SmYrbA exhibits the structural fold of BolAs with some special features. It shows a winged helix-turn-helix motif formed by four helices and a three-stranded β-sheet (α1β1β2η2α3α3′β3 in which η: 3_10_-helix) (Figure 1A and Supplementary Figure S1A). As SmYrbA belongs to the BolA_H subfamily, the [H/C]-loop (loop connecting the β1-β2 strands) contains a histidine (His^32^) that is located on the N-terminal side of the β2-strand, as seen in other BolA_H atomic structures. The invariant phenylalanine (Phe^42^) from η2 takes part in the central hydrophobic core that seems essential for BolAs [18]. A significant difference is the position of the invariant histidine (His^67^). SmYrbA contains an additional α3′-helix in which His^67^ is located whereas in other BolA structures this residue is positioned at the N-terminal extremity of the β3-strand (Supplementary Figure S1B). Although this results in quite different Cα-atom positions (mean distance of 6 Å) when compared with other BolA structures, the positions of the side chains remain close (mean distance of 3 Å for the Nε2-atoms).

The most important feature in these structures is the ability of SmYrbA to incorporate nickel, copper or cobalt atoms as clearly demonstrated by crystallographic refinements (Figure 1B) but also by X-ray fluorescence spectrometry (Figure 1C). The Co, Cu and Ni metal ions are coordinated in an octahedral geometry, bound to His^32^ and His^67^ of SmYrbA and four water molecules (Figure 1A,B). Within this octahedral coordination, the His^32^ and His^67^ residues and the water molecules number 5 and 6 define the equatorial plane whereas the two other water molecules (3 and 4) form two axial bonds (Figure 1B,D). In SmYrbA–Co and SmYrbA–Ni structures, the metal atom is almost equidistant from its ligands with a metal–ligand distance of 2.3 and 2.1 Å, respectively (Figure 1D). In the case of SmYrbA–Cu, the four equatorial bonds (2.0 Å) have the same length and are shorter than those observed in SmYrbA–Ni/Co whereas the two axial bonds (2.4 Å) are both elongated (Figure 1D). This difference is due to the Jahn–Teller effect in crystals of hexacoordinated copper(II) complexes [57]. It is noticeable that a lithium atom present in the crystallization condition was modeled into the electron density of the high-resolution structures of SmYrbA–Ni and SmYrbA–Cu (Figure 1A). This atom is at the same position in both structures and is almost equidistant (approximately 1.9 Å) from its ligands (Oδ1-Asp^25^, O-Leu^26^, Oδ1-Asp^29^ and O-His^32^), with a tetrahedral geometry. All these residues belong to the [H/C]-loop, but both Asp and Leu residues are not or poorly conserved in YrbA/BolA proteins (Supplementary Figure S1).

Although we have been unable to obtain a structure for an apo-SmYrbA, we sought to investigate whether a structural rearrangement occurs upon metal binding. A slight but significant difference in the far-UV visible CD spectra between SmYrbA and copper acetate-treated SmYrbA was visible at approximately 188 and 219 nm (Supplementary Figure S2). While it is not possible to firmly define what are the changes, they most likely occur in the region where the histidine metal ligands are. Comparing the structure of the metal-bound SmYrbA with metal-free BolA-like protein structures found in the Protein Data Bank, we tentatively attributed the changes to the shortening of the α3-helix and the formation of the additional α3′-helix only visible in SmYrbA (Supplementary Figure S1B). Interestingly, a structure of a BolA_H from the pathogen *Coxiella burnetii* ligating a Co atom was solved in the frame of a structural genomic initiative for drug design, deposited in the Protein Data Bank but never discussed [58]. An octahedral coordination is visible for the Co atom. Both conserved histidine residues (equivalent to His^32^ and His^67^) act as ligands together with three water molecules and another histidine located in the [H/C]-loop from a symmetry-related molecule (Supplementary Figure S3). The superimposition of *C. burnetii* BolA_H and SmYrbA structures highlights the similarity of the complexes despite the different positions of the invariant histidines (N-terminal end of β3-strand for *C. burnetii* BolA_H and α3′-helix for SmYrbA). The fact that there is no α3′-helix in *C. burnetii* BolA_H does not support a crucial and universal function of the α3′-helix seen in SmYrbA for BolA metalation.

**Figure 2:**
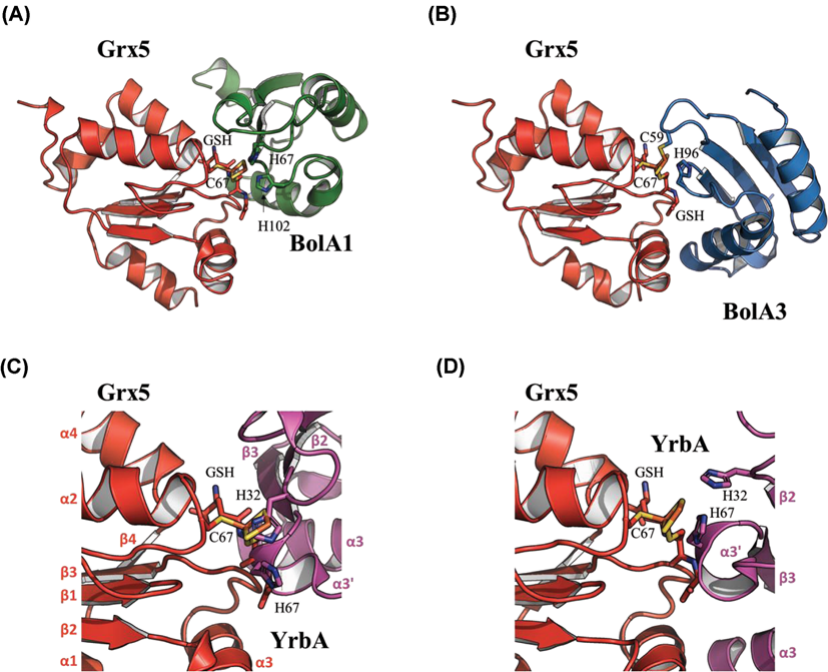
Superposition of SmYrbA on to the structural models of human BOLA1–GRX5 and BOLA3–GRX5 holo-heterodimers Structural models of [2Fe–2S] BOLA1–GRX5 (**A**) and of [2Fe–2S] BOLA3–GRX5 (**B**) obtained by molecular docking from NMR mapping [21]. SmYrbA was superimposed on BolA homologs in BOLA1–GRX5 (**C**) and in BOLA3–GRX5 (**D**). GRX5, BOLA1, BOLA3 and SmYrbA structures are in red, green, blue and purple, respectively. The invariant C-terminal histidine residue (His^67^, His^96^ or His^102^ in SmYrbA, BOLA3 or BOLA1, respectively), conserved residues present in the [H/C]-loop (His^32^, Cys^59^ or His^67^ in SmYrbA, BOLA3 or BOLA1, respectively), the catalytic cysteine Cys^67^ in GRX5 and GSH involved in Fe–S cluster binding are shown.

The two conserved histidine residues of BolA_H are assumed to participate in the binding of the iron atom in [2Fe–2S]-cluster bridged BolA_H-Grx heterodimers [13,14,18,22,23,25]. We superimposed SmYrbA structure on BolA monomer present in [2Fe–2S] BOLA1–GRX5 and [2Fe–2S] BOLA3–GRX5 complexes. These models have been obtained with human proteins in which the orientation of BOLA with respect to GRX5 was different in BOLA1–GRX5 and BOLA3–GRX5 heterodimers (Figure 2A,B) [21]. In both cases, the superimposition results in a steric clash between the glutathione bound to GRX5 and the singular α3′-helix of SmYrbA (Figure 2C,D). Thus, either a conformational change of SmYrbA α3′-helix is required when interacting with SmGrx2, or both proteins adopt an original arrangement compatible with the formation of SmYrbA–SmGrx2 heterodimer.

### SmYrbA binds specific divalent metal ions

Owing to the above observations, the binding kinetics and parameters of various metal ions on SmYrbA were investigated by ITC and the switchSENSE biosensor technology. Both methods allow determining the dissociation constants (*K*_d_) but the first (Supplementary Figure S4) gives indications about the thermodynamic parameters of the reaction and the second (Supplementary Figure S5) gives access to the association and dissociation (*k*_on_, *k*_off_) rates. We obviously included Ni^2+^, Co^2+^, Cu^2+^ metal ions in these experiments but also Zn^2+^ given its propensity to be bound by histidyl residues, Fe^3+^ and Fe^2+^ given the described properties of BolA-like proteins to provide His ligands for Fe–S cluster binding in heterodimers with Grx, and Ca^2+^ as a negative control (Table 2, Supplementary Figures S4 and S5). Interactions were detected with Ni^2+^, Co^2+^ and Cu^2+^, but also Zn^2+^ and Fe^2+^ whereas no interaction was detected with Fe^3+^ and Ca^2+^. In all cases, the best fit for ITC data was obtained with a one-site fit curve indicating that SmYrbA possesses only one binding site for each of these metal ions as observed in the X-ray structures.

**Table 2:** Interaction of SmYrbA with SmGrx2 and metal ions

	SmGrx2	Ni^2+^	Cu^2+^	Zn^2+^	Fe^2+^	Co^2+^	Ca^2+^
ITC							
*n*	1.27 ± 0.03	1.25 ± 0.01	0.98 ± 0.01	1.10 ± 0.02	1.19 ± 0.47	1.04 ± 0.09	-
*K*_d_ (µM)	6.8 ± 0.7	10.4 ± 0.4	36.2 ± 2.5	21.7 ± 1.1	33.9 ± 7.4	58.8 ± 7.1	-
ΔH (kcal.mol^−1^)	−2.9 ± 0.1	−11.3 ± 0.1	−17.0 ± 0.3	−8.0 ± 0.2	−12.2 ± 5.3	−6.1 ± 0.7	-
–TΔS (kcal.mol^−1^)	−0.3	0.4	0.9	0.1	0.5	0.0	-
ΔG (kcal.mol^−1^)	−3.2	−11.0	−16.1	−7.9	−11.7	−6.1	-
switchSENSE							
*k_on_* (M^−1^.s^−1^)	2410 ± 200	23400 ± 5700	15100 ± 1000	17800 ± 2800	ND	3190 ± 800	-
*k_off_* (s^−1^)	0.014 ± 0.003	0.606 ± 0.065	0.044 ± 0.011	0.562 ± 0.038	ND	0.671 ± 0.061	-
*K*_d_ (µM)	5.6 ± 1.3	25.9 ± 6.9	2.9 ± 0.7	31.6 ± 5.5	ND	210.3 ± 56.0	-

**Figure 3:**
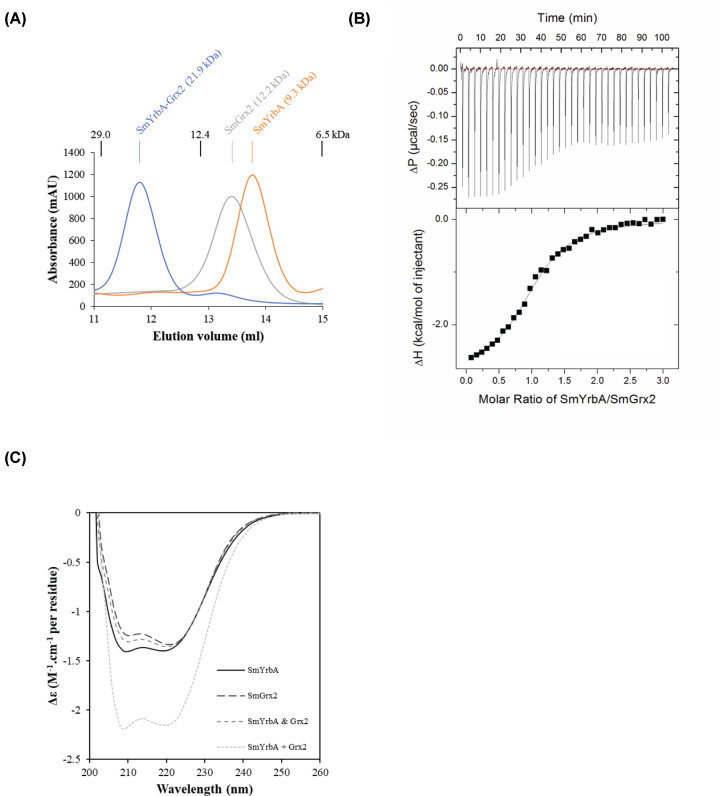
*S. meliloti* YrbA interacts with glutaredoxin 2 (**A**) Representative elution profile on analytical size-exclusion chromatography (Superdex™ 75 10/300 column) for SmYrbA (orange), SmGrx2 (gray) and a mixture of SmYrbA and SmGrx2 (blue). Absorbance at 280 nm was recorded. The respective apparent molecular masses (9.3, 12.2 and 21.9 kDa) have been estimated from the calibration curve established using proteins with elution volume of 29, 12.4 and 6.5 kDa (see ‘Experimental’ section). Considering the respective theoretical molecular masses of 7.9 and 12.1 kDa for SmYrbA and SmGrx2, the data indicate a monomeric oligomerization state for SmYrbA and SmGrx2 and the formation of a heterodimer when both proteins are mixed. (**B**) Raw ITC data (top panels) and binding isotherm data (bottom panels) for SmYrbA titration with SmGrx2. (**C**) Structural rearrangements upon complex formation assessed by far-UV CD spectroscopy. The CD spectra of SmYrbA, of SmGrx2, and of SmYrbA and SmGrx2 before (SmYrbA and SmGrx2) and after (SmYrbA + SmGrx2) mixing the cuvette contents are shown. Δε values were calculated based on the concentration and the number of residues of SmYrbA and SmGrx2 monomers.

The analysis of SmYrbA–ligand binding reactions by ITC and switchSENSE is presented with standard errors. The thermograms and isotherms of titration corresponding to experiments performed with SmGrx2 are presented in Figure 3B, those performed with divalent metal ions in Supplementary Figure S4. Results from the kinetics analysis of SmYrbA-ligand interaction determined using switchSENSE are presented in Supplementary Figure S6 (SmGrx2) and Supplementary Figure S5 (divalent metal ions). Abbreviations: *n*, number of binding sites; ND, not determined; −TΔS, change in entropy; ΔG, Gibbs free energy; ΔH, change in enthalpy.

An interaction was observed using both ITC and switchSENSE methods with *K*_d_ values in the same range for the Co^2+^, Ni^2+^, Cu^2+^ and Zn^2+^ metal ions (Table 2). Although there are some differences in the *K*_d_ values between both methods, the poorest affinity was observed for Co^2+^ ions in both cases. On the one hand, these data may explain the lower X-ray diffraction quality of SmYrbA-Co crystals. On the other hand, they raise the question why we could not co-crystallize SmYrbA with Zn^2+^ ion. In all cases, the binding is exothermic (change in enthalpy (ΔH) from −17.0 to −6.1 kcal.mol^−1^) and exergonic (Gibbs free energy (ΔG) from −16.1 to −6.1 kcal.mol^−1^). The comparable values of enthalpy and Gibbs energy changes show that the binding of Co, Ni, Cu and Zn metals is strongly enthalpy driven. Among the existing protein–ligand binding models, these thermodynamic values would be in agreement with an induced-fit model [59]. The conformation changes upon metal ion coordination could correspond to the α3′-helix folding (see above).

Because BolA-like proteins provide two ligands for Fe–S cluster binding in heterodimers with Grx, the ability of SmYrbA to interact with Fe^2+^ and Fe^3+^ was also investigated but only by ITC method because these experiments required to use anaerobically prepared samples and short-time experience. A *K*_d_ value of 33.9 ± 7.4 µM was determined for Fe^2+^ (Table 2 and Supplementary Figure S4) whereas no interaction was detected with Fe^3+^. This is rather surprising since SmYrbA should in principle bind Fe^3+^ in the context of the Fe–S cluster-bound heterocomplex with SmGrx2. The thermodynamic parameters (ΔG, ΔH and change in entropy (−TΔS)) are in the range of those determined for Ni^2+^, Co^2+^, Cu^2+^ and Zn^2+^. Hence, the reason why no SmYrbA–Fe^2+^ complex could be crystallized may be linked to iron oxidation in the crystallization solution.

### SmYrbA interacts with SmGrx2

To analyze the capacity of SmYrbA to interact with SmGrx2, both apo-proteins were incubated, and the resulting mixture analyzed by analytical size-exclusion chromatography and compared with the behavior of individual proteins. While SmYrbA and SmGrx2 existed as monomeric forms as attested by the presence of a single peak with deduced apparent molecular masses of 9.3 and 12.2 kDa, respectively, a major peak with an estimated apparent molecular mass of 21.9 kDa was obtained when both proteins were mixed together suggesting the formation of an heterodimer (Figure 3A). The binding stoichiometry was then evaluated by ITC. The obtained thermogram was fitted to a one-site binding model (*n*=1.27 ± 0.03) indicating the formation of a heterocomplex at a 1:1 ratio (Figure 3B). A *K*_d_ of 6.8 ± 0.7 µM was deduced from these measurements (Table 2 and Figure 3B). In parallel, real-time measurements of binding kinetics (*k*_on_, *k*_off_) and affinities (*K*_d_) were also achieved using the switchSENSE. After forming a His-tagged SmYrbA–dsDNA conjugate, the association and following dissociation of SmGrx2 were accurately determined from the changes in fluorescence of the DNA-associated fluorochrome (Supplementary Figure S6). From the sensorgrams (Supplementary Figure S6), a slow association (*k*_on_: 2410 ± 200 M^−1^.s^−1^) and a fast dissociation (*k*_off_: 0.014 ± 0.003 s^−1^) between SmYrbA and SmGrx2 were determined (Table 2). Based on the dissociation rate constant, a dissociation halftime of approximately 50 s (*T*_1/2_ = ln 2/*k*_off_) was obtained in these conditions, indicating a probable transient complex. From the binding kinetics, it is also possible to obtain a *K*_d_ = *k*_off_/*k*_on_ of 5.6 ± 1.3 µM, a value similar to the one obtained by ITC (Table 2). These *K*_d_ values are in the range of those previously described for complexes involving other BolA_H members as AtBOLA1–GRXS14, EcBolA–Grx4 and EcYrbA–Grx4, ranging from 3.4 to 7.0 µM [18,23]. In complexes involving BolA_C members, such as the yeast Fra2–Grx3 and the human BOLA2–GRX3, the affinity appears relatively weaker with *K*_d_ values ranging from 20.8 to 30.5 µM [19,22,23]. This difference of affinity in complexes involving BolA_H or BolA_C members may eventually point to distinct roles as already hypothesized [18,20,21,23].

Interestingly, the thermodynamic parameters determined by ITC (Table 2) indicate that the association reaction of both partners is a spontaneous process. As observed for the Gibbs free energy of binding (ΔG = –3.2 kcal.mol^−1^), for the enthalpy (ΔH = –2.9 kcal.mol^−1^) and for the entropy (–TΔS = –0.3 kcal.mol^−1^), the association is both enthalpically and entropically driven with good hydrogen bonding and favorable conformational changes. This result is consistent with the one obtained in previous studies performed by NMR spectroscopy which indicated that the interaction of BolAs and Grxs from plants is electrostatically driven [18].

To further characterize SmYrbA–SmGrx2 interaction, we used far-UV CD spectroscopy making use of a quartz spare split-compartment CD cuvette (Figure 3C). CD spectra were recorded from 200 to 260 nm for the proteins alone and before and after mixing the contents of two compartments filled with SmYrbA and SmGrx2, respectively. Mixing of the proteins in a 1:1 stoichiometry resulted in a modified CD spectrum in the 205–225 nm region (Figure 3C). This indicates that changes in the secondary structure contents of the proteins occur upon complex formation. The protein regions and residues cannot be precisely mapped here but previous NMR studies have already shown that interfacing residues do not involve Fe–S cluster ligands [18–22]. As already determined, almost all interfacing residues are in β2/β3-strands and in α3-helix on the BolA side. On the Grx side (αβαβαββαα topology), it is somewhat more complicated as NMR chemical shift mapping showed perturbations in most of the secondary structure elements. However, based on distribution of residues on the surface of Grxs, the interfacing area involves α2-helix and the region between helices α3 and α4 [18–22]. So, structural alterations upon SmYrbA–SmGrx2 heterodimer formation visible by CD should take place in these areas.

### Involvement of SmYrbA and SmBolA in iron homeostasis

**Figure 4:**
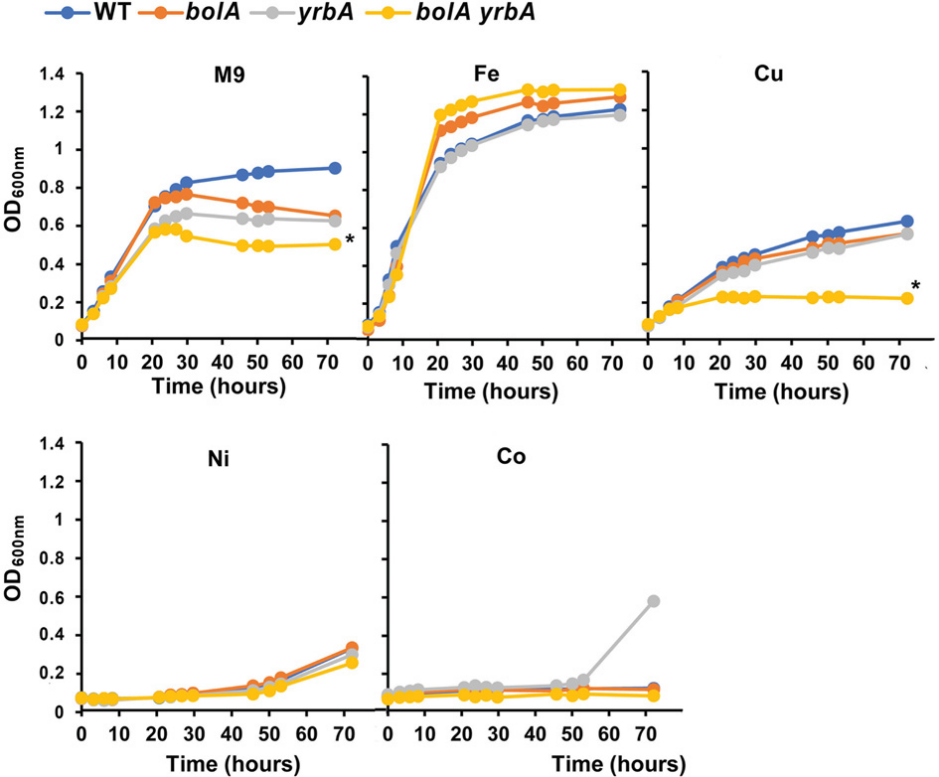
Growth curves of *S. meliloti* strains in the presence of metals The growth of free-living Rm2011 wildtype strain, *bolA, yrbA* and *bolA yrbA* mutant strains was monitored in M9 medium supplemented or not with Fe (1 mM), Cu (0.5 mM), Ni (0.5 mM) and Co (0.5 mM) by measuring the OD_600 nm_. The data are representative of three independent experiments. The asterisk (*) indicates a significant difference (*P*≤0.05) using Student’s *t* test on the last time point of the growth curve.

To analyze the physiological importance of SmYrbA in *S. meliloti*, we constructed a mutant strain inactivated for the *yrbA* gene. In addition, considering that a certain redundancy may arise from the presence of the SmBolA paralog, insertion mutant strains inactivated in the *bolA* gene or in both genes (*bolA yrbA* double mutant) were also isolated*.* We analyzed the effect of these mutations on the bacterial growth in M9 minimal medium supplemented or not with the metals which were shown to bind to SmYrbA, i.e., Fe, Cu, Ni, Co (Figure 4). First, the growth of all mutant strains (*bolA, yrbA* and *bolA yrbA*) was slightly impaired in M9 minimal medium compared with the *S. meliloti* wt Rm2011 strain, the double mutant being slightly more affected than the two single mutants. Interestingly, supplementing the medium with 1 mM Fe improved the growth of the wt strain and no more growth difference was observed with the mutant strains. For other metals, contrasted effects have been observed. The addition of Co (0.5 mM) and Ni (0.5 mM) strongly impaired the growth of all strains while they did not affect their growth at 0.1 mM (not shown). For Cu (0.5 mM), the growth of the four strains was decreased as compared with the growth in M9, but the *bolA yrbA* mutant was more affected. Overall, the results showed that supplementation of a minimal medium with iron, but not with other metals, allowed restoring the growth of the *bolA, yrbA* and *bolA yrbA* mutant strains. Altogether, these results provide a physiological evidence for a connection of SmYrbA (and SmBolA) with iron homeostasis in *S. meliloti*. The effect seen with copper may be because it affects Fe or Fe–S cluster binding by the BolA or BolA–Grx complexes. Indeed, several reports indicated that copper toxicity in bacteria is connected to interference with Fe–S cluster proteins and to their assembly processes [60,61].

**Figure 5:**
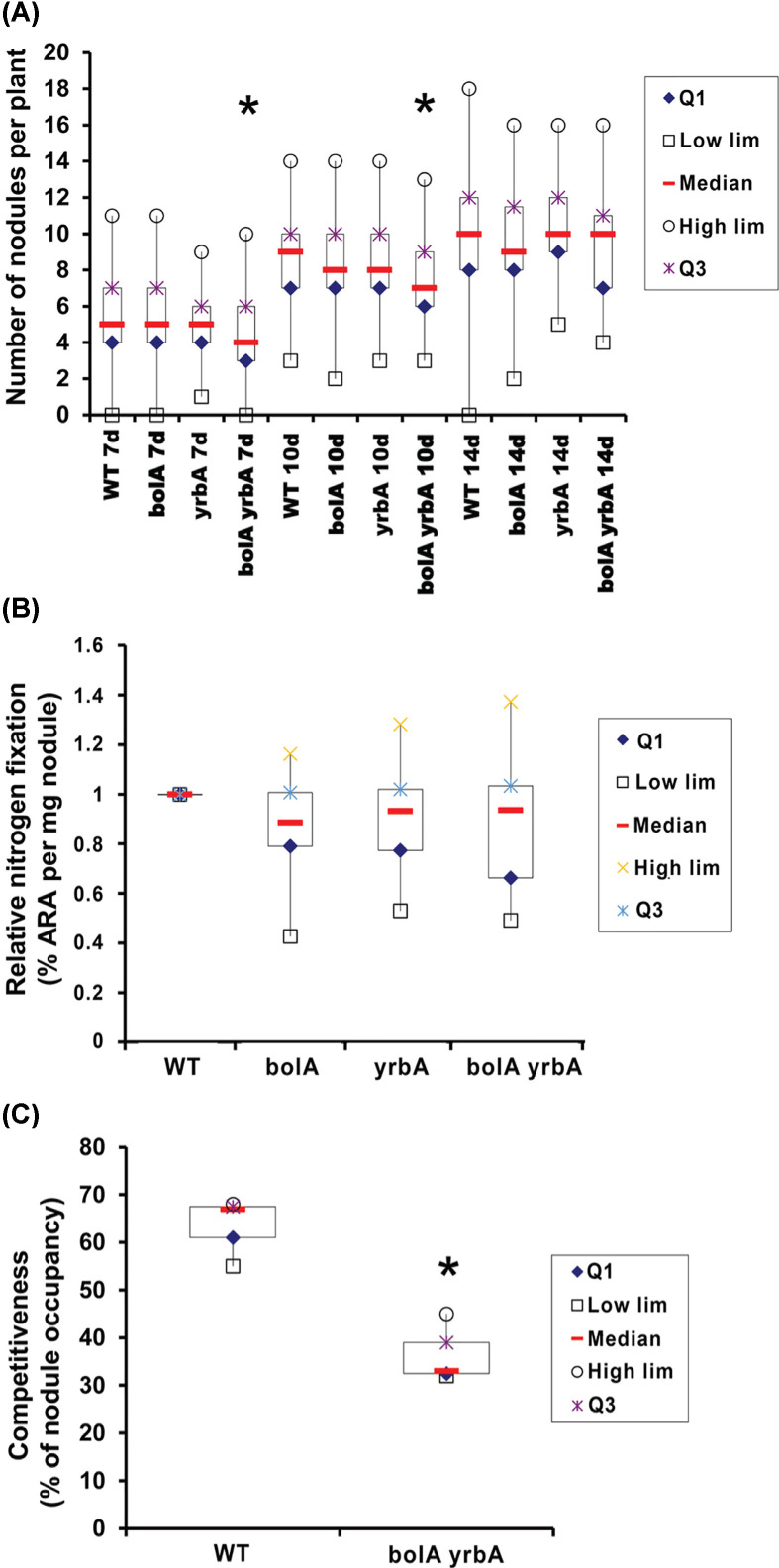
Nodulation efficiency and nitrogen-fixing capacities of the *bolA* and *yrbA* mutant strains (**A**) Plants were inoculated with the Rm2011 wildtype strain or with the *bolA, yrbA, bolA yrbA* mutant strains. The data shown represent the number of nodules per plant at 7, 10 and 14 days post-inoculation for three independent experiments. (**B**) The acetylene reduction assay (ARA) was carried out at 21 dpi. The data shown are the values per milligram of nodule for three independent experiments. (**C**) The competition assay was performed with the Rm2011 wildtype strain and the *bolA yrbA* mutant strain. The asterisks (*) indicate a significant difference (*P*≤0.05) using Student’s *t* test.

Having shown that SmGrx2 is required for an optimal symbiotic nitrogen fixation by participating to the regulation of iron metabolism [40], the capacity of the *bolA, yrbA* and *bolA yrbA* mutant strains to nodulate and produce nitrogen-fixing nodules was examined by *M. truncatula* inoculation experiments. The nodulation and nitrogen fixation efficiencies and competition capacity were analyzed to define the symbiotic capacities of the mutant strains (Figure 5). No significant difference was observed in the nitrogen fixation efficiencies of *bolA* and *yrbA* mutant strains compared with *S. meliloti* wt strain Rm2011 strain. In contrast, the *bolA yrbA* mutant strain showed a significantly delayed nodulation compared with Rm2011 strain. This result was reinforced by the significantly lower competitiveness of the *bolA yrbA* mutant strain compared with the wt strain. In conclusion, mutations in *bolA* and *yrbA* impaired the efficiency of the first stages of nodulation and decreased the competitiveness for nodulation but the nitrogen-fixing efficiency is *in fine* not significantly affected once nodulation is established.

## Conclusions

Various activities and roles for BolA proteins have been proposed from recent investigations conducted in prokaryotes (mainly *E. coli*) and eukaryotes (*S. cerevisiae, A. thaliana*, human), i.e*.* endonuclease, stress-responsive transcriptional regulator of the cellular morphology, and either regulator of iron transcription factors or maturation factor for Fe–S proteins. Whereas the connection with Grx for the Fe homeostasis regulatory function seems clear in fungi, other BolA functions may be independent of Grx. Here, although we have observed an interaction between SmYrbA and SmGrx2 that might be important for some cellular functions such as, for instance, RirA regulation, the difference between *grx2* and *bolA yrbA* mutant phenotypes suggests that SmBolA/SmYrbA and SmGrx2 likely perform different functions in the nodulation context. This makes echo to the differences observed in yeast regarding the role of mitochondrial orthologs in Fe–S protein maturation [20,30].

A role of SmYrbA (and SmBolA) in Fe homeostasis seems obvious from the growth restoration effect observed for *bolA yrbA* double mutant strain upon addition of iron in minimal medium. However, the observed binding of several other divalent metals (Co, Ni, Cu and Zn) by SmYrbA and the observed growth defects of *bolA yrbA* double mutant strain in minimal medium but also its sensitivity to copper may point to a more general regulatory role of YrbA in the cross-talk between metals. It is well documented that an excess in one transition metal perturbs the homeostasis of other metals. In support of this idea, a metatranscriptomic study of metal-contaminated soils identified BolA proteins as novel actors of metal resistance [62]. The overexpression of identified BolA genes in specific yeast mutants conferred resistance to diverse metals (Zn, Co, Cd and Mn). This is however accompanied by a specific increase in the intracellular iron concentration. Since SmYrbA binds metals via two histidine residues that are otherwise involved in Fe–S cluster ligation in complex with Grx, we propose that metal binding by bacterial YrbA (and maybe more generally by BolA_H representatives) is thus important for regulating the known interplay between Fe homeostasis and other metals such as Zn, Cd or Co.

## Supplementary Material

Supplementary Figures S1-S6 and Supplementary Table S1

## Data Availability

Coordinates and structure factors of SmYrbA–Co, SmYrbA–Cu and SmYrbA–Ni were deposited in the Protein Data Bank under accession codes 5NFL, 5NFM and 5NFK, respectively.

## Abbreviations

ΔG: Gibbs free energy
ΔH: change in enthalpy
CD: circular dichroism
Fe–S: iron–sulfur
Grx: glutaredoxin
IPTG: isopropyl β-D-1-thiogalactopyranoside
ITC: isothermal titration calorimetry
*K*_d_: dissociation constant
*k*_off_: dissociation rate constant
*k*_on_: association rate constant
−TΔS: change in entropy
