# Supplementary material for: Sinorhizobium meliloti YrbA binds divalent metal cations using two conserved histidines
Source: Biosci Rep. 2020 Oct 6;40(10):BSR20202956. doi: 10.1042/BSR20202956 (PMC7538681; doi:10.1042/BSR20202956)
Supplement: Supplementary Figures S1-S6 and Supplementary Table S1 [file BSR-2020-2956_supp.pdf]

Supplementary information to the paper « *Sinorhizobium meliloti YrbA binds diverse divalent metal cations using two conserved histidines*” by Roret et al

**SmYrbA-Fw**<br>**SmYrbA-Rv**	CCCCCCCCATATGGCACCAGGCGAT<br>CCCCGGATCCTTACTCCGGCGCGCT	pET-12a, pET-15b
**SmGrx2-Fw**<br>**SmGrx2-Rv**	CCCCCCATGGCTAGCGGAATCAACGATTTCATC<br>CCCCGGATCCTCAGGCGGCGCCCTTGACGGA	pET-3d

Table S1. Primers used to amplify SmYrbA and SmGrx2

The NdeI (CATATG), BamHI (GGATCC) and NcoI (CCATGG) restriction sites are underlined. Fw: forward; Rv: reverse.

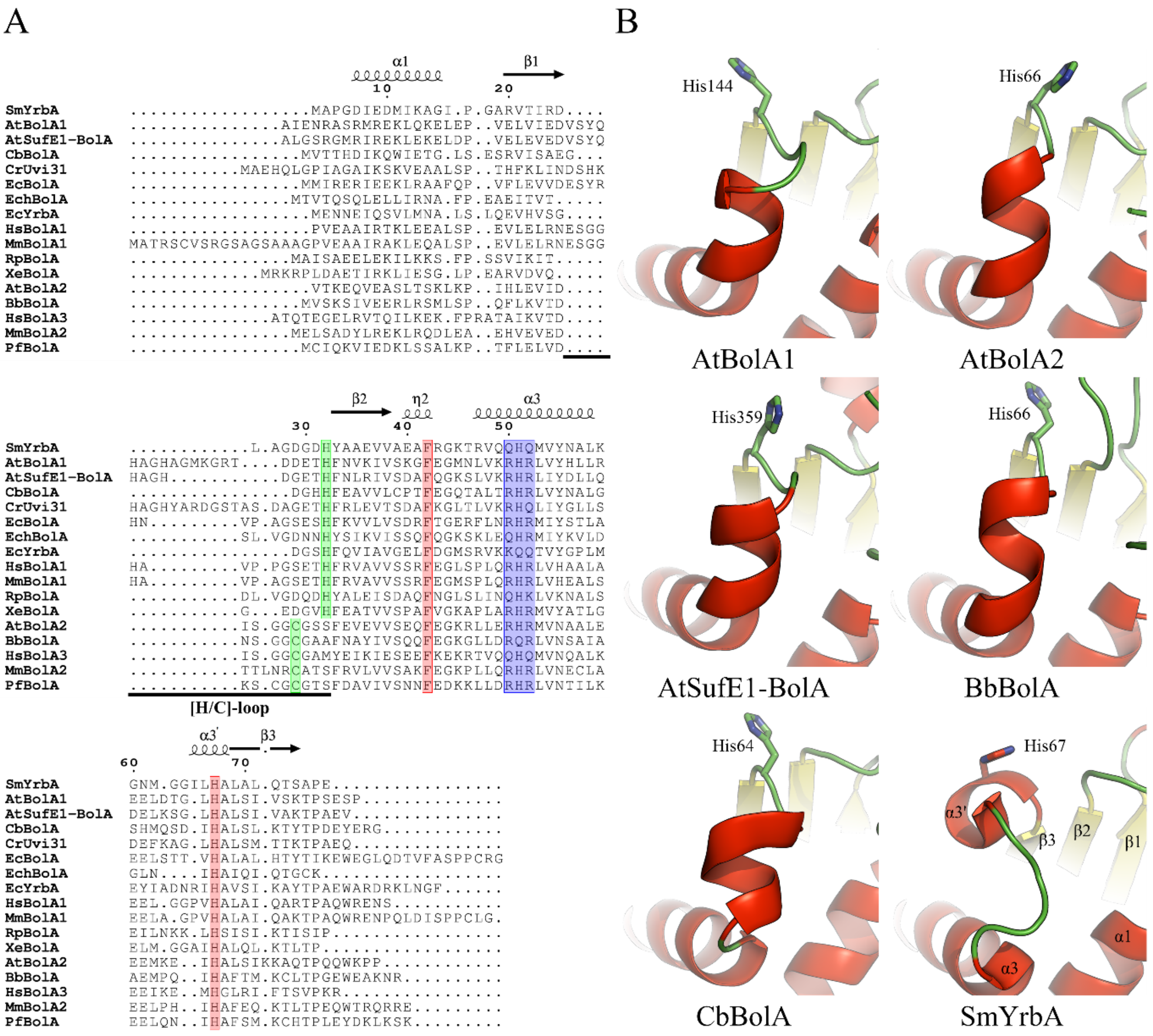

Figure S1. Structural and multiple sequence alignment of BolA proteins

(A) Multiple sequence alignment of BolA proteins present in the PDB. SmYrbA secondary structures are plotted on top of the alignment. The invariant Phe42 and His67 are highlighted in red. The conserved histidine (BolA_H) and cysteine (BolA_C) residues present in the [H/C]-loop between β1 and β2 strands are colored in green. The RHR motif is pointed in blue. The abbreviations used are At: *Arabidopsis thaliana*; Bb: *Babesia bovis*; Cb: *Coxiella burnetii*; Cr: *Chlamydomonas reinhardtii*; Ec: *Escherichia coli*; Ech: *Ehrlichia chaffeensis*; Hs: *Homo sapiens*; Mm: *Mus musculus*; Pf: *Plasmodium falciparum*; Rp: *Rickettsia prowazekii* and Xe: *Xanthomonas euvesicatoria* [1–7]. (B) Structural alignment of BolA proteins. For clarity, only the area around the invariant His67 is shown. The coordinates of *A. thaliana*, *B. bovis,* and *C. burnetii* BolA proteins are from previous crystal structures (PDB entries 4PUG, 4PUH, 4PUI, 3O2E and 3TR3).

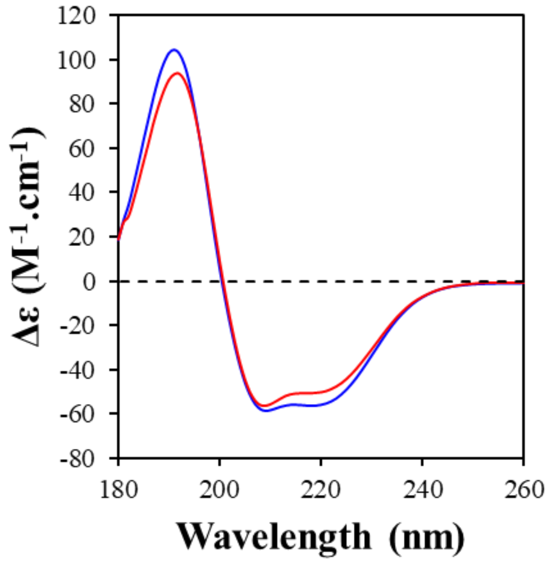

Figure S2. Structural changes in SmYrbA upon metal binding

Changes in the Far-UV CD spectrum of SmYrbA upon copper binding. After dialysis against a 30 mM Tris-HCl pH 8.0, 1 mM EDTA buffer to remove trace amount of metal ions and then against MilliQ water, the spectrum of SmYrbA (81 µM) was recorded in the absence (blue line) or presence (red line) of 3 mM copper acetate. Δε value was calculated based on the concentration of SmYrbA.

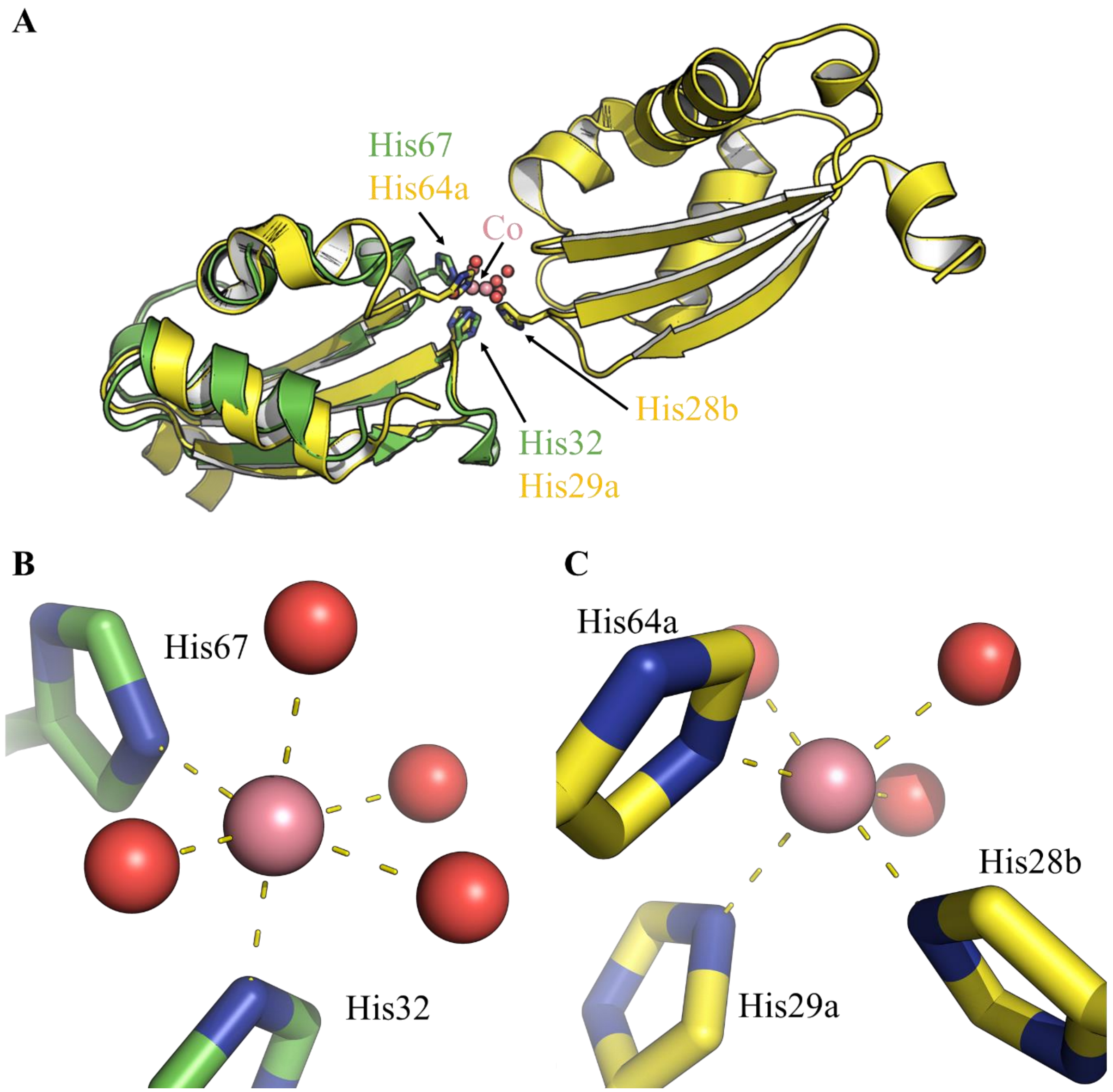

Figure S3. Superposition of two X-rays structures of BolA-Co complexes

(A) Superposition of *S. meliloti* YrbA (green, PDB entry: 5NFL) and *C. burnetii* BolA (yellow, PDB entry: 3TR3). Both proteins are in complex with a cobalt atom. (B) The coordination of Co^{2+} with His32 and His67 of SmYrbA and four water molecules. (C) The coordination of Co^{2+} with His29a and His64a of CbBolA, His29b of a symmetric molecule and three water molecules [6]. Cobalt ions and water molecules are shown as pink and red spheres, respectively.

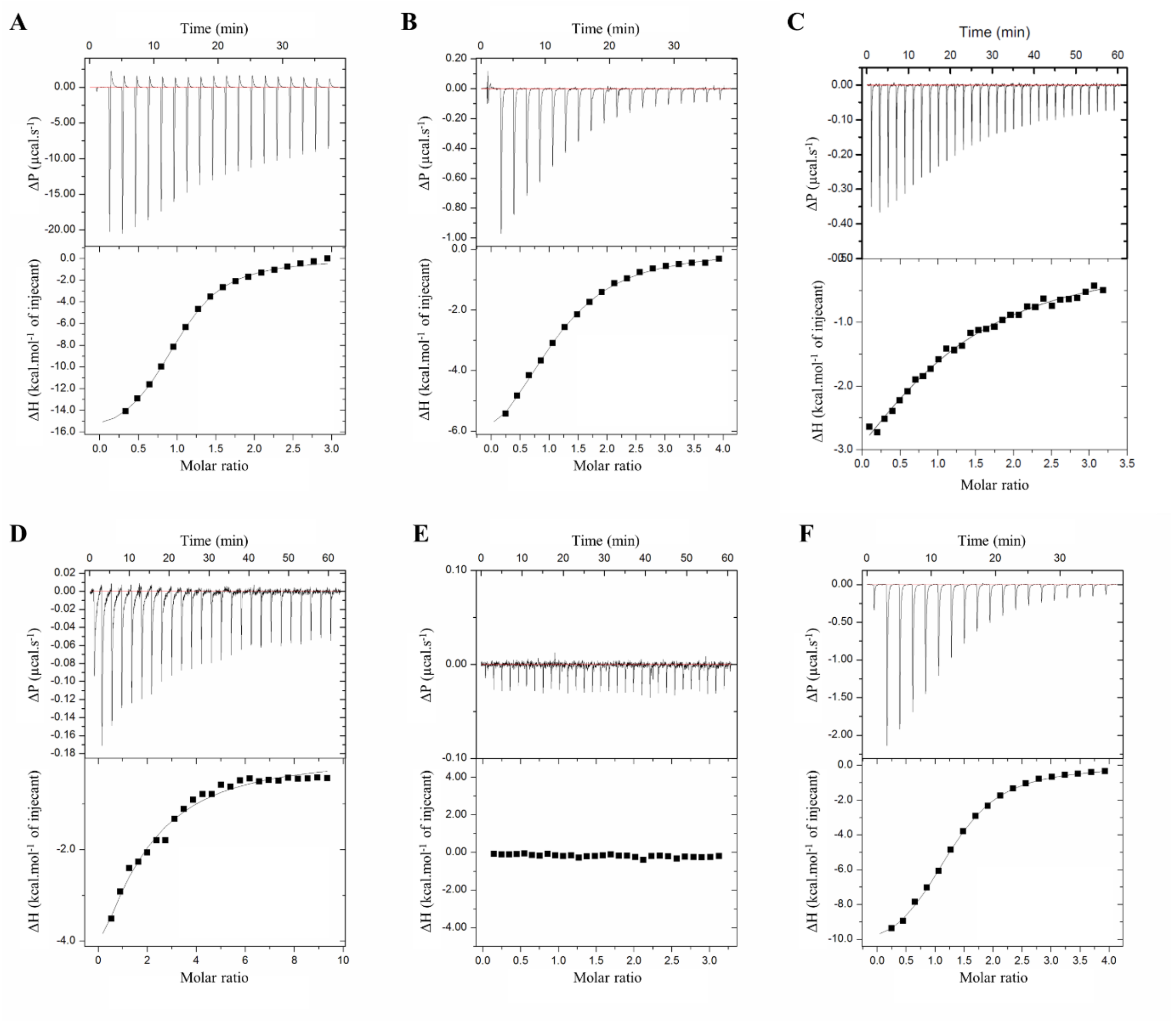

Figure S4. Isothermal titration calorimetry of interaction of metal ions with *S. meliloti* YrbA

Titrations curves (top) were acquired at 25°C using 50 µM SmYrbA and titrating 1 mM of (A) Cu acetate, (B) Zn acetate, (C) $CoCl_2$, (D) $FeSO_4$, (E) $CaCl_2$ and (F) $NiCl_2$. Data were fitted using a "one-binding-site" mode (bottom curve) with ORIGIN software.

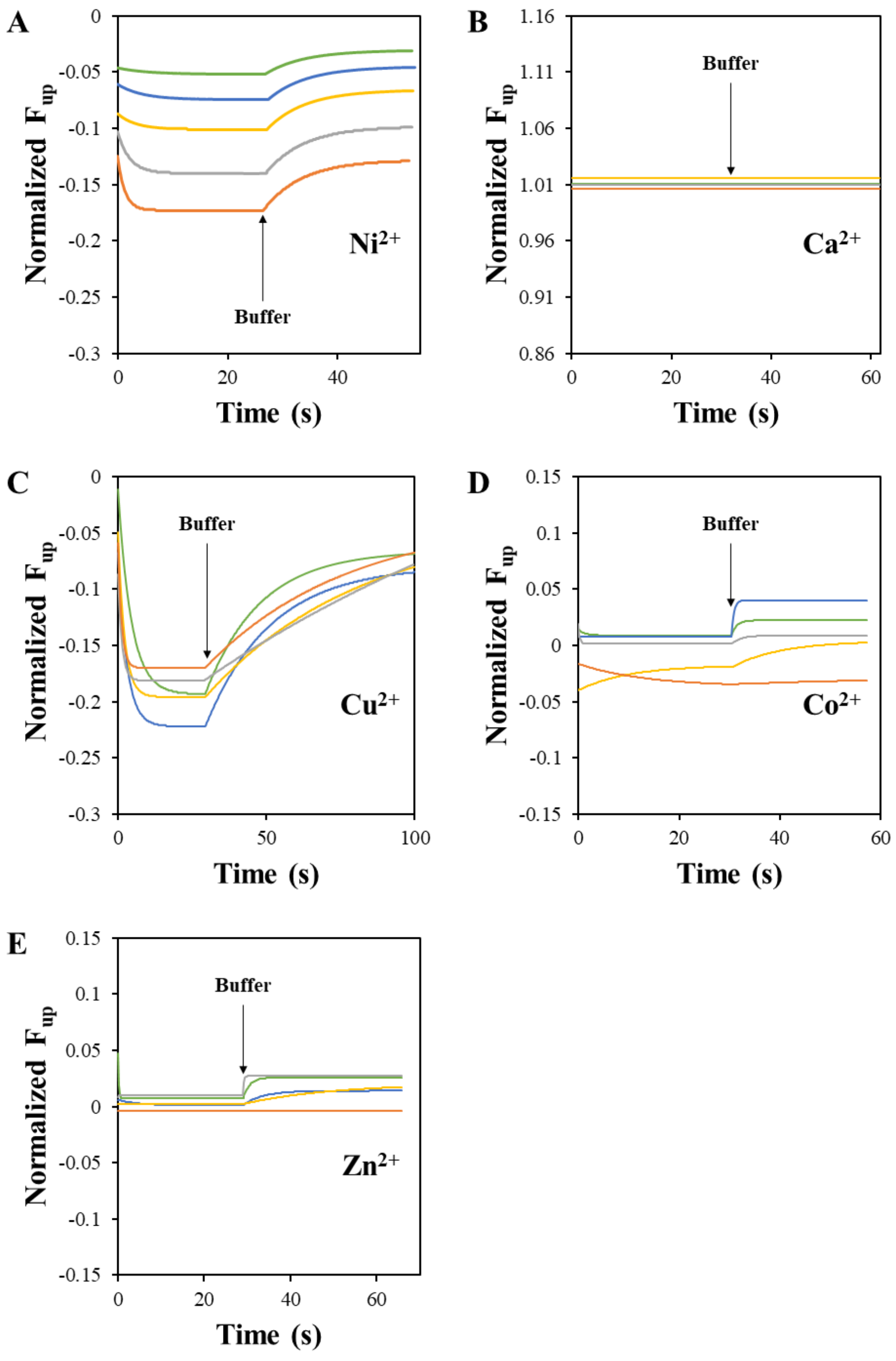

Figure S5. Real-time switchSENSE® analysis of interactions of SmYrbA with different metal ions

switchSENSE® kinetics analysis of SmYrbA bound to DNA nanolever and interacting with increasing concentrations of $NiCl_2$ (**A**), $CaCl_2$ (**B**), Cu acetate (**C**), $CoCl_2$ (**D**) and Zn acetate (**E**). the concentrations used are 6.25 (green), 12.5 (blue), 25 (yellow), 50 (grey) and 100 µM (orange). Exponential fits of the upward switching fluorescence responses (F_{up}) are shown. Dissociation kinetics were determined with metal-free buffer.

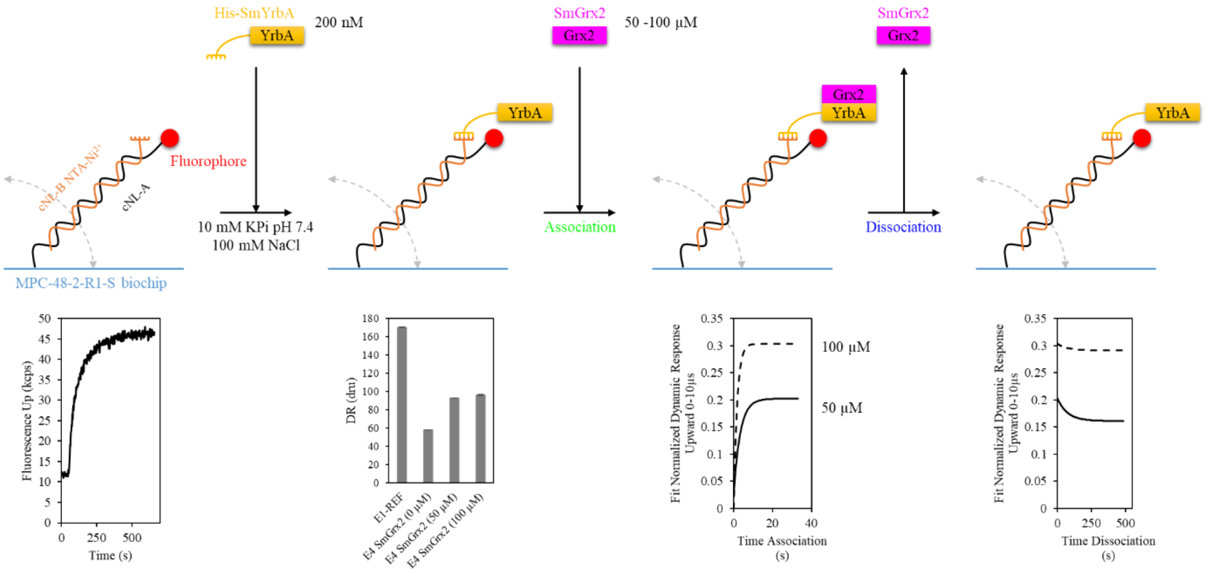

Figure S6. Real-time switchSENSE® analysis of interaction between SmGrx2 and SmYrbA

Top: Schematic of an NTA_3-tagged DNA nanolever capturing a His-tagged SmYrbA in the presence of Ni^{2+} and association/dissociation of SmYrbA-SmGrx2 complex. cNL-A and cNL-B: complementary nanolever with the nucleotidic sequence A and B. KPi: potassium phosphate.

Bottom (experimental data): The first curve shows the fluorescence response for the hybridization of the NTA_3-tagged single strain cDNA onto the nanolever. The histogram shows the dynamic response (DR) of electrode 4 (E4) in the presence of the captured His-tagged YrbA and, then, in the presence of increasing concentrations of SmGrx2 (50 and 100 µM), comparatively to a reference electrode (E1-REF) free of protein. The two last graphs show the association and dissociation kinetics determined from exponential fits of the dynamic responses (DR values evaluated between 0 and 10 µs). F_{up}: Time-resolved fluorescence response during upward switching; kcps: kilocounts per second; dru: dynamic response unit.

References

[1] T. Kasai, M. Inoue, S. Koshiba, T. Yabuki, M. Aoki, E. Nunokawa, E. Seki, T. Matsuda, N. Matsuda, Y. Tomo, M. Shirouzu, T. Terada, N. Obayashi, H. Hamana, N. Shinya, A. Tatsuguchi, S. Yasuda, M. Yoshida, H. Hirota, Y. Matsuo, K. Tani, H. Suzuki, T. Arakawa, P. Carninci, J. Kawai, Y. Hayashizaki, T. Kigawa, S. Yokoyama, Solution structure of a BolA-like protein from Mus musculus., Protein Sci. 13 (2004) 545–548.

[2] K.H. Chin, F.Y. Lin, Y.C. Hu, K.H. Sze, P.C. Lyu, S.H. Chou, NMR structure note--solution structure of a bacterial BolA-like protein XC975 from a plant pathogen Xanthomonas campestris pv. campestris., J Biomol NMR. 31 (2005) 167–172.

[3] J. Abendroth, A.S. Gardberg, J.I. Robinson, J.S. Christensen, B.L. Staker, P.J. Myler, L.J. Stewart, T.E. Edwards, SAD phasing using iodide ions in a high-throughput structural genomics environment., J Struct Funct Genomics. 12 (2011) 83–95.
[4] T. Roret, P. Tsan, J. Couturier, B. Zhang, M.K. Johnson, N. Rouhier, C. Didierjean, Structural and spectroscopic insights into BolA-glutaredoxin complexes, J. Biol. Chem. 289 (2014) 24588–24598. https://doi.org/10.1074/jbc.M114.572701.
[5] G.W. Buchko, A. Yee, A. Semesi, P.J. Myler, C.H. Arrowsmith, R. Hui, Solution-state NMR structure of the putative morphogene protein BolA (PFE0790c) from Plasmodium falciparum., Acta Crystallogr F Struct Biol Commun. 71 (2015) 514–521.
[6] M.C. Franklin, J. Cheung, M.J. Rudolph, F. Burshteyn, M. Cassidy, E. Gary, B. Hillerich, Z.K. Yao, P.R. Carlier, M. Totrov, J.D. Love, Structural genomics for drug design against the pathogen Coxiella burnetii., Proteins. 83 (2015) 2124–2136.
[7] M.A. Uzarska, V. Nasta, B.D. Weiler, F. Spantgar, S. Ciofi-Baffoni, M.R. Saviello, L. Gonnelli, U. Mühlenhoff, L. Banci, R. Lill, Mitochondrial Bol1 and Bol3 function as assembly factors for specific iron-sulfur proteins, Elife. 5 (2016). https://doi.org/10.7554/eLife.16673.
